# Selective Ligand Recognition by a Diversity-Generating Retroelement Variable Protein

**DOI:** 10.1371/journal.pbio.0060131

**Published:** 2008-06-03

**Authors:** Jason L Miller, Johanne Le Coq, Asher Hodes, Roman Barbalat, Jeff F Miller, Partho Ghosh

**Affiliations:** 1 Department of Chemistry and Biochemistry, University of California San Diego, La Jolla, California, United States of America; 2 Department of Microbiology, Immunology, and Molecular Genetics, David Geffen School of Medicine at UCLA, University of California Los Angeles, Los Angeles, California, United States of America; 3 The Molecular Biology Institute, University of California Los Angeles, Los Angeles, California, United States of America; 4 Section of Molecular Biology, University of California San Diego, La Jolla, California, United States of America; Howard Hughes Medical Institute, California Institute of Technology, United States of America

## Abstract

Diversity-generating retroelements (DGRs) recognize novel ligands through massive protein sequence variation, a property shared uniquely with the adaptive immune response. Little is known about how recognition is achieved by DGR variable proteins. Here, we present the structure of the *Bordetella* bacteriophage DGR variable protein major tropism determinant (Mtd) bound to the receptor pertactin, revealing remarkable adaptability in the static binding sites of Mtd. Despite large dissimilarities in ligand binding mode, principles underlying selective recognition were strikingly conserved between Mtd and immunoreceptors. Central to this was the differential amplification of binding strengths by avidity (i.e., multivalency), which not only relaxed the demand for optimal complementarity between Mtd and pertactin but also enhanced distinctions among binding events to provide selectivity. A quantitatively similar balance between complementarity and avidity was observed for *Bordetella* bacteriophage DGR as occurs in the immune system, suggesting that variable repertoires operate under a narrow set of conditions to recognize novel ligands.

## Author Summary

The immune system long has been considered unique in its capacity to recognize alien molecules. This anticipatory recognition depends on a repertoire of receptors—antibodies and T cell receptors—with binding sites capable of accommodating trillions of different amino acid sequence combinations. A similar capacity was discovered recently in diversity-generating retroelements (DGRs), which are encoded by prokaryotes and the bacteriophages that infect them. The receptor-binding protein Mtd of *Bordetella* bacteriophage is encoded by a DGR and accommodates trillions of sequences in its binding site. DGR-programmed variation of Mtd allows the phage to use alternative host receptors—an ability that is crucial for phage infection because the expression pattern of these host receptors changes according to the environmental status of the bacterium. We sought to understand how Mtd recognizes receptors by determining the structure of an Mtd variant bound to the host receptor pertactin. With this structural knowledge, we then compared the binding properties of different variants and their ability to support phage infection. Despite large differences in physical details between Mtd and immunoreceptors, general principles emerged through which variable repertoires achieve selective molecule recognition, providing a view of how the Mtd family can rapidly evolve novel specificities.

## Abstract 3

Although the massively variable protein Mtd of the*Bordetella* bacteriophage diversity-generating retroelement recognizes ligands in a different manner from antigen receptors of the immune system, general principles underlying anticipatory recognition are strikingly conserved.

## Introduction

The vertebrate adaptive immune response long has been considered unique among biological systems in having the capacity to recognize novel ligands in an anticipatory fashion [1]. Such anticipatory recognition depends on antigen receptors that accommodate massive sequence variation in their ligand binding sites, which enables these receptors to have vastly diverse binding specificities. In the immune system of jawed vertebrates, antibodies and T cell receptors (TCRs) have ligand binding sites that are estimated to accommodate ∼10^14–16^ potential amino acid sequences [2]. Likewise, in the immune system of jawless vertebrates, lymphocyte receptors have binding sites that are estimated to accommodate ∼10^14^ potential sequences [3]. Recently, the first instance outside the immune system of massive sequence variability directed towards anticipatory recognition of novel ligands was discovered in diversity-generating retroelements (DGRs) [4].

DGRs have been identified in the genomes of 21 prokaryotic organisms and bacteriophages to date [5] (M. Gingery and J.F.M., unpublished data). The most extensively characterized DGR is encoded by *Bordetella* bacteriophage, which is capable of producing ∼10^13^ potential sequences in the variable protein major tropism determinant (Mtd) [4–7]. Mtd is a pyramid-shaped trimeric protein [7] that localizes to the ends of phage tail fibers and functions as the phage's receptor-binding protein [4,6]. DGR-programmed variation of Mtd enables the phage to use alternative *Bordetella* receptors for infection [4]. This is essential to the phage because the expression pattern of *Bordetella* surface molecules (i.e., potential phage receptors) changes according to the environmental status of the bacterium [4,8,9].

**Figure 1:**
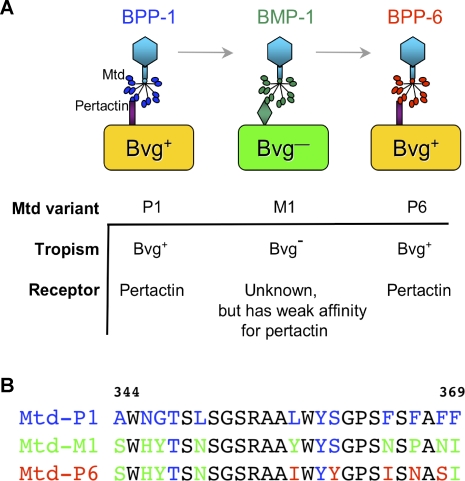
Tropism and Receptor Specificities of *Bordetella* Bacteriophages (A) The Mtd-P1 variant is expressed by BPP-1 phage and sets the tropism of the phage to Bvg^+^ *Bordetella* due to recognition of the Bvg^+^-specific receptor pertactin. The Mtd-M1 variant is a direct descendant of Mtd-P1 and is expressed by BMP-1 phage. It sets tropism to Bvg^–^ *Bordetella* through recognition of an unidentified receptor. Mtd-M1 also binds pertactin, albeit nonproductively. The Mtd-P6 variant is a direct descendant of Mtd-M1 and is expressed by BPP-6 phage. It sets the tropism of BPP-6 to Bvg^+^ *Bordetella* due to recognition of pertactin. (B) Variable region sequences of Mtd-P1, Mtd-M1, and Mtd-P6. Variable residues are in color, and invariant residues in black. For Mtd-P1, variable residues are in blue. For Mtd-M1, variable residues in common with Mtd-P1 are in blue, and those unique to Mtd-M1 are in green. For Mtd-P6, residues in common with Mtd-M1 or Mtd-P1 are in green or blue, respectively, and those unique to Mtd-P6 are in red. Mtd has 381 residues in total.

Environmentally influenced gene expression patterns in *Bordetella* are regulated by the BvgAS two-component system, with virulence factors being expressed exclusively in the in vivo pathogenic or Bvg^+^ phase and genes required for motility being expressed exclusively in the ex vivo or Bvg^–^ phase (Figure 1) [8–10]. Other genes are expressed preferentially at intermediate levels of Bvg activation [11]. The Bvg^+^-specific adhesin pertactin [12,13] is the only Mtd receptor whose identity is currently known [4,7] (Figure 1A), but the existence of Bvg^–^-specific and other receptors has been demonstrated genetically [4].

**Figure 2:**
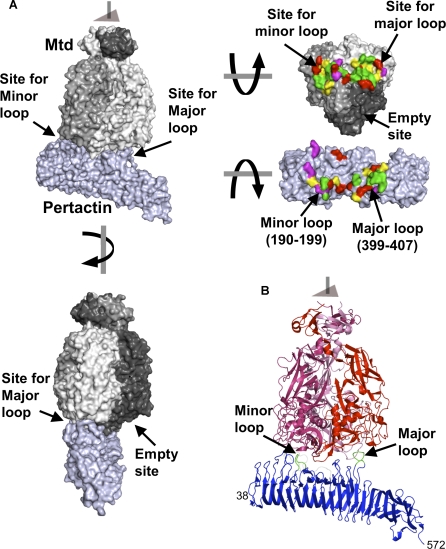
Structure of Mtd-P1 Bound to Prn-E. (A) (Top left) The Mtd-P1·Prn-E complex is depicted in molecular surface representation, with each protomer in the Mtd-P1 trimer colored a different shade of gray and Prn-E colored blue. The trimer axis of Mtd is depicted by a gray triangle at the top. One site of trimeric Mtd binds the major pertactin loop (399–407); a second site binds the minor pertactin loop (residues 190–199); the third site is empty. (Bottom left) Arrow designates a 90° rotation, which exposes the empty site to view. (Top right) Arrows designate 90° rotations (Mtd rotated back toward page, Prn-E forward toward viewer) that expose the interacting surfaces to view (green, hydrophobic contacts; red, hydrophilic contacts; yellow, hydrophobic buried; purple, hydrophilic buried). Contact residues are those having an interatomic distance between Mtd-P1 and Prn-E of ≤4 Å, and buried residues are those having an interatomic distance of >4 Å and excluding water due to association. (B) The Mtd-P1·Prn-E complex is depicted in ribbon representation (Mtd protomers in red, pink, and purple and Prn-E in blue). Prn-E loops that contact Mtd-P1 are green and labeled. The trimer axis of Mtd is depicted by a gray triangle at the top. For reference, the RGD loop of pertactin is shown in light gray in the conformation observed for B. pertussis pertactin [12] but was not modeled in the structure of the Mtd-P1·Prn-E complex.

Variation in Mtd is focused on 12 adenine-encoded amino acids that are fixed in position and scattered across its C-terminal region (Figure 1B). This pattern of variation is governed by the template-based and adenine-specific mechanism of DGR mutagenesis [4]. The 12 variable residues are organized into receptor-binding sites by the C-type lectin fold of Mtd. There are three such independent receptor-binding sites in trimeric Mtd, and these sites are located at the bottom of the pyramid-shaped trimer (Figure 2 and Figure S1) [7]. The backbone conformations of the receptor-binding sites in Mtd are notably static to sequence variation. Two factors appear to contribute to maintaining the static nature of these sites. The first is trimeric assembly of the β2β3 loop of the C-type lectin fold, which braces the binding site of a neighboring protomer (Figure S1). The second is the presence of two insert regions, which surround and also brace the binding site [7]. Sequence and structural evidence indicates that other DGRs are likely to accommodate massive sequence variation using the C-type lectin fold as well [5,7].

While the mode of antigen recognition by antibodies and TCRs is well studied, no knowledge of how a DGR variable protein recognizes its ligands is available. To address this, we determined the structure of the Mtd-P1 variant [7] bound to the Bvg^+^-specific receptor pertactin. Mtd-P1 is expressed by BPP-1 phage (Figure 1), and interaction between Mtd-P1 and pertactin is required for infection by this phage [4]. This interaction is sufficient for infection, as Bvg^–^ *Bordetella* is rendered permissive to BPP-1 infection through ectopic expression of pertactin [4].

With structural knowledge in hand, we pursued the question of why the relatively weak interaction (3 μM dissociation constant, *K*_d_) between Mtd-P1 and pertactin supports infection [7], whereas a slightly weaker interaction between Mtd-M1 and pertactin does not (Figure 1). Mtd-M1 is a direct descendant of Mtd-P1 that binds pertactin [7] but is incapable of using pertactin productively as a receptor for infection; it instead uses an unidentified receptor expressed by Bvg^–^ *Bordetella* [4]. By comparing properties of these two Mtd variants as well as of Mtd-P6, a pertactin-dependent revertant of Mtd-M1 that closely resembles Mtd-M1 in sequence (Figure 1), we identified avidity (i.e., multivalency) as an essential determinant of selective infection. We found that avidity not only served to expand the number of productive interactions but also enhanced discrimination among interactions. We suggest that these same principles contribute to the robust and selective response of sequence variable repertoires in the vertebrate adaptive immune system.

## Results

### Recognition of Pertactin by Mtd-P1

We isolated, crystallized, and determined the structure of Mtd-P1 (120 kDa per trimer) [7] bound to the 60 kDa ectodomain of B. bronchiseptica pertactin (Prn-E; residues 38–640). Pertactin is a β-helix protein that localizes to the *Bordetella* outer membrane and belongs to the autotransporter family [12,13]. The 60 kDa ectodomain of pertactin is proteolytically processed from a 93 kDa precursor and remains tightly but noncovalently associated with the bacterial cell surface [14,15]. Structure determination to 3.16 Å resolution revealed that a single Mtd-P1 trimer, although possessing three independent receptor-binding sites, associated with just one Prn-E molecule (Figure 2). The 3:1 Mtd-P1/Prn-E stoichiometry is consistent with measurements made in solution [7]. Two independent copies of the ∼180 kDa Mtd-P1·Prn-E complex occupied the asymmetric unit of the crystal (Table S1). Except for certain unbound loops of pertactin, molecular details were unambiguous and nearly identical in the two Mtd-P1·Prn-E complexes. No large conformational changes occurred in Mtd-P1 upon binding [7], and while the structure of free B. bronchiseptica Prn-E is not known, comparison to the structure of the closely related free B. pertussis Prn-E [12] suggested no large conformational changes occurred in pertactin upon binding.

A total of ∼2300 Å^2^ was buried at the Mtd-P1·Prn-E interface. This large figure compares favorably with the 1680 Å^2^ of an average antibody–antigen interface [16]. The majority of the Mtd-P1·Prn-E surface (66%) was composed of hydrophobic residues, which is consistent with prior characterization of this association as being entropically driven [7]. About three-fourths of the buried surface area came from residues that were in atomic contact (≤4 Å), and the remaining from residues that were not in contact but close enough to have excluded water (Figure 2 and Tables S2 and S3).

**Figure 3:**
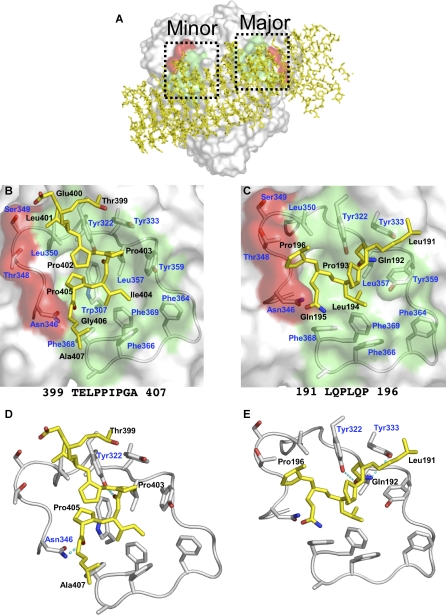
Mtd-P1 Receptor-Binding Sites (A) The Mtd-P1·Prn-E complex is depicted with Mtd-P1 in molecular surface representation and Prn-E in yellow stick representation. The view is looking at the base of the pyramid-shaped Mtd trimer. The surfaces of the Mtd sites that bind the major and minor pertactin loops are colored (green, hydrophobic residues; red, hydrophilic residues). (B) Contacts between Mtd-P1 and the major pertactin loop. Mtd-P1 is depicted in molecular surface representation, as in (A). Backbone atoms of the Mtd-P1 variable region are depicted in gray curvilinear representation, and side chain carbons, oxygens, and nitrogens in gray, red, and blue, respectively. Mtd-P1 residues are labeled in blue. The major loop of Prn-E (residues 399–407, sequence below panel) is depicted in stick representation (backbone in yellow and side chain carbons, oxygens, and nitrogens in yellow, red, and blue, respectively). Prn-E residues are labeled in black. The backbone carbonyls of Prn-E Pro403 and Pro405 are also depicted. (C) Contacts between Mtd-P1 and the minor pertactin loop, depicted as in (B). (D and E). Hydrogen bonds to (D) major and (E) minor pertactin loops are shown as cyan dashed lines. Atom coloring is as in panels (B) and (C).

Two loops of pertactin emanating from its β-helix scaffold contacted Mtd. These loops nestled separately into two of the three receptor-binding sites of Mtd (Figures 2 and 3). The third receptor-binding site of Mtd was unoccupied and sterically occluded from binding another Prn-E molecule. One of the contacting pertactin loops, composed of residues 399–407, formed the majority of the interactions with a total of ∼870 Å^2^ being buried at this interface and was called the “major loop”. The other loop, composed of residues 190–199, made less extensive but nevertheless essential interactions (see below), with a total of ∼585 Å^2^ being buried at this interface, and was called the “minor loop”.

**Figure 4:**
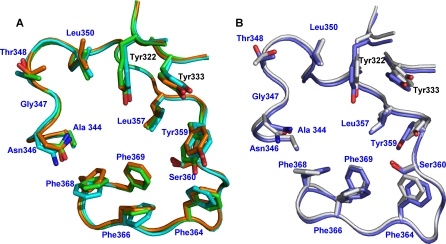
Superposition of Mtd-P1 Variable Regions (A) Mtd-P1 sites from the Mtd-P1·Prn-E complex that bind the major (orange) or minor (green) pertactin loops or no pertactin loop (cyan) are superposed (average root-mean-square deviation 0.303 Å for 376 Cα atoms). The backbone is shown in curvilinear representation, and side chains of variable residues, along with the invariant residues Tyr322 and Tyr333, are shown in stick representation. Variable residues are labeled in blue, and invariant ones in black. (B) The Mtd-P1 site from the Mtd-P1·Prn-E complex that binds the major pertactin loop (gray) is superimposed with a site from unbound Mtd-P1 (blue) (root-mean-square deviation 0.450 Å for 376 Cα atoms).

The receptor-binding sites in Mtd-P1 consisted of shallow pockets that contained a central cluster of aromatic residues and a stripe of polar residues along one edge (Figure 3B and 3C). Relatively planar interfaces also occur in antibody–protein complexes [17]. Aromatic residues of Mtd dominated the interactions with both the major and the minor loops of pertactin. The variable Mtd residues Tyr359, Phe366, and Phe368 and the invariant Mtd residues Tyr322 and Tyr333 were particularly significant in terms of buried surface area (Table S2). Tyrosines are also dominant at antibody–antigen interfaces, forming ∼25% of these contacts [18]. The invariant Mtd residues Tyr322 and Tyr333 are located on the β2β3 loop, which infiltrates into the binding site from a neighboring protomer (Figure S1) [7]. The involvement of these β2β3 loop tyrosines in binding reinforced the notion that the trimeric nature of Mtd was essential for function [7]. Although the three separate and independent binding sites of Mtd-P1 interacted with different chemical environments, they were strikingly indistinguishable in conformation from one another and from unbound Mtd-P1 (Figure 4).

Pertactin buried mostly hydrophobic residues (Leu401, Pro403, Ile404, and Pro405) from the major loop 399–407 within the Mtd aromatic cluster in one binding site and Leu191, Gln192, and Leu194 from the minor loop 190–199 in a second binding site (Figure 3B and 3C and Table S3). The hydrogen bonding capacity of pertactin Gln192 in the minor loop appeared to be satisfied by the invariant Mtd residues Tyr322 and Tyr333 (Figure 3E). In addition, it appeared that two hydrogen bonds were formed to the main chain carbonyl atoms of the major loop (Figure 3D).

The major and minor pertactin loops have no obvious sequence relationship to each other, and unlike other regions of pertactin, neither loop is antigenically variant [19]. The conformations of the major and minor loops in their free state are not known, but these and other pertactin loops are likely to be highly flexible, as deduced from the structure of free B. pertussis pertactin [12]. On the basis of this evidence, we suspect that the major and minor loops of B. bronchiseptica pertactin probably differ in conformation between bound and free states (Figure S2). Residues interacting with Mtd-P1 are conserved between B. bronchiseptica and B. pertussis, except for Pro403, which is substituted by Ser. The substitution was tolerated, as Mtd-P1 bound B. pertussis Prn-E (unpublished data).

Most notably, the shape fit between Mtd and pertactin resembled an antibody–antigen complex as quantified by surface complementarity (SC) [20]. Complexes that have undergone evolutionary fine-tuning have high SC values of 0.70–0.74 (e.g., 0.77 for the interprotomer interfaces of Mtd, where 1.0 is perfect complementarity), whereas antibody–antigen interfaces have lower SC values of 0.64–0.68, reflecting their anticipatory and non-coevolved fit. Mtd-P1 bound the major and minor loops with SC values of 0.67 and 0.62, respectively, which distinguished these interactions as antibody-like. The small adjustments seen in side chain positions of Mtd-P1 upon binding were important for achieving this shape fit (Figure 4B), as documented by the lower SC values of 0.55 and 0.56 for free Mtd-P1 modeled in complex with the major and minor loops, respectively.

### Differences between Productive and Nonproductive Interactions with Pertactin

We next pursued the question of why interaction between Mtd-P1 and pertactin leads to infection but interaction between Mtd-M1 and pertactin does not (Figure 1). Mtd-M1 is a direct descendant of Mtd-P1 known to bind pertactin, but unlike Mtd-P1, this interaction is nonproductive and does not lead to infection [4,7]. Exactly 9 of 12 variable residues differ between Mtd-P1 and Mtd-M1, with most of the aromatic cluster in Mtd-P1 substituted by polar residues, as shown by direct structural comparison [7] (Figure 1B and Figure S3). These nine changes are sufficient to switch specificity from pertactin to an unknown receptor expressed exclusively by Bvg^–^ *Bordetella* [4], possibly glycans of the O-antigen as implicated by genetic evidence (unpublished data).

**Figure 5:**
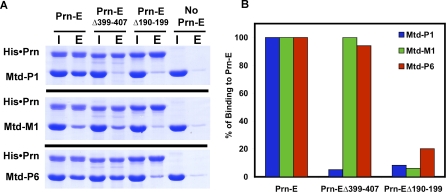
Dependency of Mtd Interactions on Major and Minor Pertactin Loops (A) Coprecipitation of 60 μM (trimer concentration) Mtd-P1 (top), Mtd-M1 (middle), and Mtd-P6 with His-tagged wild-type Prn-E, Prn-EΔ399–407, Prn-EΔ190–199, or no Prn-E using Ni^2+^-NTA beads and visualized by SDS-PAGE and Coomassie staining. I, relative amount incubated with Ni^2+^-NTA beads; E, relative amount eluted from beads. (B) Quantification of binding of Mtd-P1 (blue), Mtd-M1 (green), and Mtd-P6 (red) to Prn-EΔ399–407 and Prn-EΔ190–199, normalized by binding to Prn-E. Values were corrected for background binding (no Prn-E) to Ni^2+^-NTA beads.

With structural evidence having identified the 190–199 and 399–407 loops as the pertactin epitope for Mtd-P1, we asked whether Mtd-M1 shares this epitope. Major and minor pertactin loop deletion mutants were constructed, and purified His-tagged Prn-E mutants were assayed for association with Mtd-P1 in an in vitro coprecipitation assay. We found that loss of either the major (Prn-E Δ399–407) or minor (Prn-E Δ190–199) pertactin loop severely attenuated association with Mtd-P1 as compared to that of wild-type Prn-E (Figure 5A and 5B). This indicated that both major and minor loops were necessary for association with Mtd-P1 and, conversely, that each of the Mtd sites contacting pertactin conferred a portion of the affinity. In contrast, Mtd-M1 bound Prn-E Δ399–407 equally well as wild-type Prn-E (Figure 5A and 5B), which indicated that the major loop was not required for association with Mtd-M1. The minor loop 190–199 appeared to form at least part of the epitope for Mtd-M1, as deletion of this loop reduced binding substantially (Figure 5A and 5B). However, we note that interaction between Mtd-M1 and pertactin was weak and that partial loss of binding would thus be difficult to differentiate from complete loss in this assay. These results indicated that Mtd-M1 did not retain the same pertactin epitope as Mtd-P1 but fortuitously bound a separate epitope that contained, at least in part, loop 190–199.

### Evolution of Productive Interactions

To gain further insight into the selectivity of infection, we isolated pertactin-dependent revertants of Mtd-M1. The most informative revertant was Mtd-P6, which was expressed by BPP-6 phage and differed at only 5 of 12 variable residues from Mtd-M1 (Figure 1). These five changes were modeled with high confidence due to the static nature of Mtd and predicted to enlarge the hydrophobic portion of the binding site and segregate hydrophobic from polar areas (Figure S3).

As with Mtd-M1, deletion of major loop 399–407 in Prn-E did not abrogate association with Mtd-P6 (Figure 5A and 5B). Deletion of the minor loop 190–199 reduced association but not fully, which indicated that the pertactin epitope for Mtd-P6 involved loop 190–199, at least in part. This pattern closely resembled that of Mtd-M1, and due to the close sequence similarity between Mtd-M1 and Mtd-P6, it strongly suggested that these two Mtd variants had a shared pertactin epitope.

**Figure 6:**
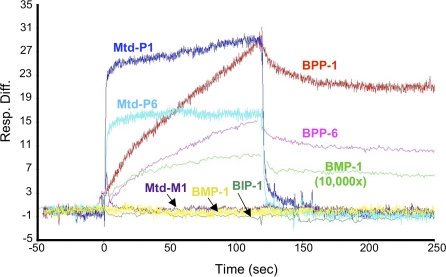
Affinity of Mtd and Avidity of Phage The SPR sensorgrams showing association of Mtd-P1 (blue), Mtd-P6 (cyan), and Mtd-M1 (purple) and of phage BPP-1 (red), BPP-6 (pink), BIP-1 (thin green), BMP-1 (yellow), and BMP-1 10,000× (thick green) with biotinylated Prn-E immobilized on a streptavidin chip. Curves were chosen for display and represent one of six (or five in the case of BMP-1 10,000×) different concentrations.

To quantify the affinity of Mtd-P6 for pertactin, we turned to surface plasmon resonance (SPR) with biotinylated Prn-E immobilized on the surface of a streptavidin chip (Figure 6 and Figure S4). First, we validated the SPR experiment by examining association of Mtd-P1 with surface-immobilized pertactin. The *K*_d_ determined by SPR for Mtd-P1·Prn-E was 3.47 ± 0.13 μM (Table 1), agreeing almost exactly with the prior isothermal titration calorimetry measurement of 3.04 ± 0.48 μM [7]. The affinity of Mtd-M1 for pertactin was too low to be detected by SPR, as had also been the case for isothermal titration calorimetry, but a coprecipitation assay provided an estimate of ∼0.5 mM for its apparent *K*_d_ (Figure S5 and Materials and Methods section). A similar value was obtained by considering limits of detection in the SPR experiment (Materials and Methods section). By comparison, the *K*_d_ of Mtd-P6 for pertactin was 5.19 ± 3.07 μM by SPR, which demonstrated that some or all of the five differences between Mtd-M1 and Mtd-P6 resulted in a ∼100-fold increase in affinity for a pertactin epitope that was likely shared between the two.

**Table 1:**
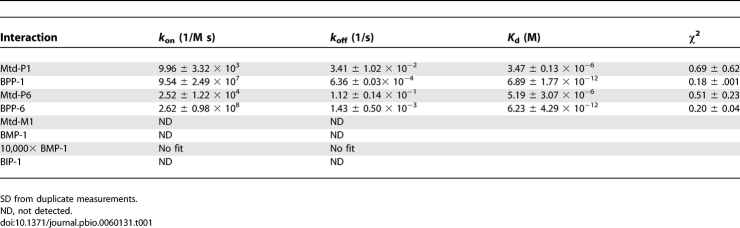
Mtd–Pertactin Interactions (SPR)

### Avidity Amplifies with Differential Gain

While these measurements looked at individual Mtd trimers, the situation is vastly different during infection. The phage has six tail fibers, and cryoelectron microscopy data suggest that each fiber has two Mtd trimers at its base [4,6] (A.H. and Z.H. Zhou, unpublished data). The existence of 12 Mtd trimers per phage raised the possibility that infection was governed by multivalent interactions between the phage and the surface-localized pertactin. To quantify how affinity between Mtd and pertactin translated into multivalent avidity [21], we turned again to SPR.

In contrast to the micromolar *K*_d_'s of Mtd-P1 and Mtd-P6 for pertactin, phage expressing these Mtd variants had dramatically amplified binding strengths, as characterized by *K*_d_'s of 6.89 ± 1.77 and 6.23 ± 4.29 pM for BPP-1 and BPP-6, respectively (Figure 6 and Table 1). This ∼10^6^-fold amplification partitioned as a ∼10^4^-fold increase in on-rate and a ∼10^2^-fold decrease in off-rate. The more prominent gains in on-rate suggested a complex and multistep association mechanism for both Mtd-P1 and Mtd-P6. This was consistent with the requirement for occupancy of two binding sites in the case of Mtd-P1 (Figure 2) and the slow on-rates of ∼10^4^ M^−1^ s^−1^ for both Mtd-P1 and Mtd-P6 (Table 1). The avidity of reovirus for mammalian cells shows a similar prominence of on-rates in enhancing binding [22]. The binding of BPP-1 and BPP-6 phage to pertactin was specific, as seen by the lack of binding by BIP-1 phage to immobilized pertactin (Figure 6). BIP-1 expresses an Mtd variant (Mtd-I1) that has no detectable affinity for pertactin [7].

Most strikingly, we observed no interaction of BMP-1 phage with pertactin, despite the fact that the Mtd-M1 had demonstrable affinity for pertactin (Figure 6 and Figure S5). However, at a ∼10^4^-fold higher concentration of BMP-1 than that used for BPP-1 or BPP-6, binding was observed (Figure 6). Binding by highly concentrated BMP-1 had a much slower on-rate than that observed for either of the other two phages but an off-rate that was apparently similar. A reliable fit to the data could not be achieved for BMP-1, but the greater concentration required to detect binding suggested an apparent multivalent *K*_d,app_ of ∼0.5 μM for BMP-1 (Materials and Methods section). These results indicated that avidity amplified binding strengths differentially. Binding strengths for Mtd-P1 and Mtd-P6 were amplified ∼10^6^-fold, but the binding strength for Mtd-M1 was amplified only ∼10^3^-fold.

### Phage Binding to *Bordetella*

**Figure 7:**
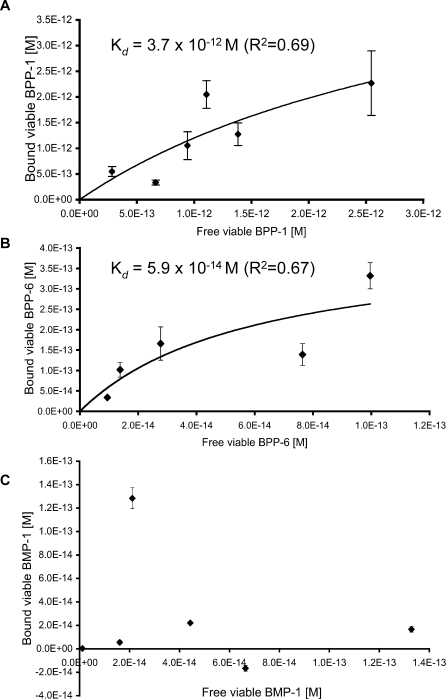
Phage Binding to *Bordetella* Binding of (A) BPP-1, (B) BPP-6, and (C) BMP-1 to Bvg^+^ *Bordetella*. For BPP-1 and BPP-6, binding to Bvg^–^ *Bordetella* was subtracted as nonspecific background, and for BMP-1, binding to E. coli was subtracted as nonspecific background. Phage concentrations were determined by plaquing on appropriate hosts and thus represent viable phage.

We lastly asked how the SPR measurements correlated with in vivo association between the phage and *Bordetella*. First, we quantified the amount of pertactin on the *Bordetella* surface to be ∼3000 molecules (Figure S6). On the basis of this, the average pertactin density on the SPR chip was estimated to be similar to that on the *Bordetella* surface (Materials and Methods section). This suggested that results from SPR measurements were applicable to understanding in vivo association. To further confirm SPR results, we directly examined binding between the phage and *Bordetella*. In contrast to the kinetic measurements of the SPR experiment, precise quantification was difficult to achieve in these equilibrium binding experiments due to limits on phage concentrations. Nevertheless these experiments provided estimates of binding strengths that confirmed the SPR results. BPP-1 and BPP-6 bound Bvg^+^ *Bordetella* tightly, with estimated *K*_d_'s in the picomolar or sub-picomolar range (Figure 7A and 7B). These values represent the specific component of binding, as binding to Bvg^–^ *Bordetella* was subtracted as nonspecific background. In contrast, specific binding of BMP-1 to Bvg^+^ *Bordetella* was too weak to be detected (Figure 7C), agreeing with the near micromolar dissociation constant suggested by SPR. This lack of binding was not due to a deficiency in the phage, as BMP-1 bound tightly and specifically to Bvg^–^ *Bordetella* (Figure S7).

## Discussion

Mtd differed strikingly from antibodies and TCRs in its mode of ligand recognition. Binding sites in antibodies and TCRs are flexible and capable of adapting to ligands through induced fit [23]. Binding sites in Mtd were instead static and associated with pertactin in a lock-and-key fashion. Despite their static nature, the binding sites in Mtd were also remarkably adaptable to different epitopes. This was seen in the sequence-unrelated major (“TELPPIPGA”) and minor loops (“LQPLQP”) of pertactin being bound by chemically and conformationally equivalent sites in Mtd-P1. Binding site adaptability is consistent with Mtd having the capacity to recognize diverse receptors and was essential for Mtd-P1 function because its association with pertactin was dependent on both major and minor loops.

The structure of Mtd-P1·Prn-E, however, did suggest some limitations. Epitopes for Mtd are likely to be restricted to portions of receptors that are flexible and therefore capable of filling the static Mtd sites. A second limitation was suggested by the pseudosymmetric mode of Mtd-P1·Prn-E association. This mode requires either a receptor large enough (>30 Å) to span multiple Mtd sites simultaneously or displayed at high enough density on the bacterial surface to do the same. Further work is needed to determine whether the pseudosymmetric mode is general to other Mtd variants and other receptors, but Mtd-P6 also appeared to bind pertactin with a 3:1 stoichiometry (unpublished data). This is consistent with pseudosymmetric association between Mtd-P6 and pertactin, but conclusive evidence awaits structure determination of this complex.

**Figure 8:**
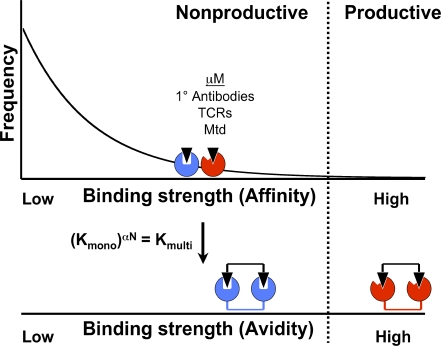
Avidity Provides Amplification and Differential Gain (Top) Distribution of variable proteins and their individual binding strengths (monovalent affinities) to a particular ligand (triangle), with two variants depicted (red and blue). (Bottom) Avidity amplifies monovalent affinities through the exponential factor α*N*, resulting in some members of the repertoire crossing a threshold required for productive interaction and biological effect. Differences between monovalent affinities are also amplified such that slightly differing monovalent affinities become vastly differing multivalent avidities.

Despite large differences in physical details between Mtd and immunoreceptors, general principles for achieving selective ligand recognition by variable repertoires emerged. Mtd was reminiscent of antibodies and TCRs in its relatively weak affinity (micromolar *K*_d_) for its ligands. This weak affinity was consistent with the suboptimal shape complementarity between Mtd and pertactin, reflecting the anticipatory encounter between these two molecules. Such anticipatory encounters contrast with coevolutionary processes that give rise to high surface complementarity and affinity [20]. Suboptimal interfaces likewise occur between (primary) antibodies and antigens and are reflected in the relatively weak affinities of antibody–antigen complexes, as characterized by micromolar *K*_d_'s [24–27]. Even lower affinity interactions, in the tens of micromolar *K*_d_ range, are observed for TCRs binding to major histocompatibility complex-peptide complexes [28]. The prevalence of micromolar *K*_d_'s suggests that high-affinity binding variants (i.e, ones that have high complementarity to their ligands) occur only rarely in variable repertoires (Figure 8).

Micromolar *K*_d_'s stand in contrast to the binding strengths required for biological effect, for example, the ∼20–50 nM *K*_d_ required for a protective antibody response [29] or picomolar *K*_d_ shown here to support *Bordetella* phage infectivity. The problem of attaining high affinity is solved by multivalent avidity [21]. For *Bordetella* phage, avidity amplified the strength of productive binding events ∼10^6^-fold, translating a micromolar *K*_d_ for the monovalent Mtd–pertactin interaction to a picomolar *K*_d_ for the multivalent phage–bacterium interaction. In the immune system, avidity has been documented to amplify monovalent interactions for immunoglobulin G's between ∼20- and 10^3^-fold and for immunoglobulin M's up to ∼10^6^-fold [24,30]. Avidity also has been observed in interactions of immunoglobulin-G-bound antigens with B cell receptors [25] and has been noted to amplify intercellular interactions between TCRs and major histocompatibility complex-peptide complexes [28,31,32]. Monomeric major histocompatibility complex-peptide complexes are unstimulatory towards T cells, in contrast to trimeric and tetrameric complexes, which constitute potent stimuli [33]. Amplification of the strength of individual binding events by avidity enables variants with suboptimal complementarity for their ligands to nevertheless achieve binding strengths required for biological effect. This results in an expansion in the number of productive interactions available to a variable repertoire (Figure 8).

For every binding event that is amplified by avidity and made productive, like that of Mtd-P1 or Mtd-P6 with pertactin, there are many more that are just slightly weaker, like that of Mtd-M1 with pertactin, and still more that are even weaker, and so on (Figure 8). Yet, what emerges from this pool of binding events with graded affinities is a highly selective response, with Mtd-P1 and Mtd-P6 supporting infectivity through pertactin but not Mtd-M1. The explanation for this lies in the differential gain of avidity.

Avidity amplifies the strength of individual binding events by the exponential factor α*N* (Figure 8) [21]. The magnitude of α*N* depends on the details of receptor density and geometry, with α being the degree of cooperativity and *N* the valency of interaction. Significantly, α*N* was ∼2 for Mtd-P1 and Mtd-P6 as well as for Mtd-M1, meaning that neither the degree of cooperativity nor the valency differed between productive and nonproductive interactions. This is consistent with Mtd-P6 and Mtd-M1 having a common pertactin epitope. Selectivity in infection instead emerged from α*N*, which effectively squared the ∼10^−6^ M *K*_d_'s of Mtd-P1 and Mtd-P6 for pertactin into the picomolar range for the phage but the ∼0.5 × 10^−3^ M *K*_d_ of Mtd-M1 only to the near micromolar range. Phage concentrations are limited to ∼10^−12^ M, explaining why a *K*_d_ in the near micromolar range for BMP-1 rendered the interaction with Bvg^+^ *Bordetella* nonproductive. Thus, avidity not only amplified the strength of individual binding events but importantly also amplified the differences between such events. A ∼100-fold difference in the micromolar *K*_d_ range for Mtd (i.e., 3–5 μM for Mtd-P1 and Mtd-P6 versus ∼500 μM for Mtd-M1) resulted in a ∼10^5^-fold difference in the picomolar *K*_d_ range for the phage (i.e., ∼6–7 pM for BPP-1 and BPP-6 versus ∼0.5 μM for BMP-1). Steps subsequent to binding may further enhance the selectivity initially provided by the differential gain of avidity [34].

The essential role of avidity in the responsiveness and selectivity of the Mtd repertoire predicts multivalency to be a general feature of variable repertories, consistent with the prevalence of multivalency in the adaptive immune system. It is striking that both Mtd and the immune system have arrived at common solutions for achieving productive interactions. In each case, only moderate surface complementarities (SC ≈ 0.65) are required for productive interactions, as the micromolar *K*_d_'s of these binding events are amplified by avidity to high affinity ranges. This amplification relaxes the demand for optimal complementarity and as a consequence expands the scope of ligands capable of being recognized, thereby making the repertoire more responsive.

The amplification effect of avidity is powerful, and it is conceivable that interactions with even worse complementarities and affinities (e.g., millimolar *K*_d_) could be made productive by avidity. For this, the exponential factor α*N* would need to be large enough to compensate for these very low affinities. However, such potent amplification would also come at a cost, creating a repertoire that is overly responsive and therefore lacking selectivity and most likely effectiveness as well (due to tendencies toward self-aggregation). It appears that this is the reason that variable repertoires have evolved to have moderate values of α*N*. Interactions by immunoglobulin G's have been documented to be amplified by α*N* values of ∼1.2, and even immunoglobulin M's, which are potentially pentavalent, have been documented to be amplified by moderate α*N* values of ∼2.5 [30]. This is similar to our results, showing that potentially dodecavalent Mtd displayed by *Bordetella* phage is amplified by α*N* of only ∼2.

These observations suggest the existence of an optimal balance between surface complementarity and avidity in variable repertoires. Moderate values in each (SC ≈ 0.65 and α*N* ≈ 2) are seen to support responsiveness and selectivity in recognition by the *Bordetella* bacteriophage DGR and the immune system. In comparison, an imbalance towards low α*N* and consequent demand for high SC would lead to sparse recognition (i.e., nonresponsiveness), and an imbalance towards high α*N* and resulting tolerance of low SC would lead to promiscuous binding (i.e., nonselectivity). Despite depending on dramatically different modes of ligand recognition, the *Bordetella* bacteriophage DGR and the immune system have evolved quantitatively similar principles for selective ligand recognition. These similarities suggest that a narrow set of conditions endow variable repertoires with responsiveness and selectivity.

## Materials and Methods

### Phage evolution.

BMP-1(Δ*brt*) lysogens were induced, as previously described [4], from B. bronchiseptica RB50 expressing *Brt* ectopically from the B. bronchiseptica filamentous hemagglutinin promoter on the pBBR1MCS-derived, medium-copy plasmid pMin1 (M. Xu and J.F. Miller, unpublished data). Resulting phage were propagated on B. bronchiseptica RB54 (Bvg^–^) and then selected for plaque formation on B. bronchiseptica RB53 (Bvg^+^) [35]. These BPP phage were screened for dependency on pertactin for infection, as assayed by their inability to form plaques on B. bronchiseptica RB50(Δ*prn*) (Bvg^+^). Pertactin-dependent phage were then lysogenized, and the variable regions of single-colony lysogens were sequenced before reinduction of phage.

Mtd variants were cloned, expressed, and purified as described previously, as was His-tagged Prn-E (residues 38–640) [7]. Loop deletions of Prn-E (Δ190–199 and Δ399–407) containing an N-terminal His tag (MGSSHHHHHHSSGLVPRGSHMAS) were constructed by strand-overlap extension PCR [36] and expressed using pET28b (Novagen). These mutant proteins were refolded from inclusion bodies and purified as previously described [7]. Size-exclusion chromatography (Superdex 200) was used to confirm the nonaggregated and monomeric state of these mutant proteins. In vivo biotinylation of Prn-E was carried out through expression of pertactin residues 38–640 carrying an N-terminal biotinylation sequence (MSGLNDIFEAQKIEWHEGAPELE) from pAN-5 (AviTag) in Escherichia coli AVB101. Biotinylated Prn-E was expressed as inclusion bodies and refolded and purified as above.

### Crystallization and structure determination.

Mtd-P1·Prn-E complexes were formed by mixing ∼1.6-fold molar excess of purified Mtd-P1 trimers with His-tagged Prn-E and separating complexes from unbound proteins using successive Ni^2+^-chelation and size-exclusion (Superdex 200) chromatographies. Protein crystals were grown by the sitting drop, vapor diffusion method at 25 °C by mixing equal volumes of 10 mg/ml Mtd-P1·Prn-E (ɛ_280calc_ 254,050 M^−1^ cm^−1^) and precipitant (2 M NaCl, 100 mM Tris, pH 8.5, 200 mM Li_2_SO_4_).

X-ray diffraction data were collected at 100 K using synchrotron radiation at the Advanced Light Source (λ = 1.000 Å; oscillation range 0.35°; beamline 5.0.1) from crystals that had been cryoprotected by soaking in precipitant solution containing 25% glycerol. Crystals of Mtd-P1·Prn-E belong to space group *P*6_1_ and contain two Mtd-P1·Prn-E complexes in the asymmetric unit. Diffraction data were processed and scaled using HKL2000 [37], and the structure was determined by the molecular replacement technique using Amore [38] and Mtd-P1 as the search model. The model was built and refined using O [39] and Refmac 5.2 [38], respectively. Noncrystallographic symmetry restraints were used in initial stages of refinement but removed in later stages, whereupon translation–libration–screw (TLS) refinement was used with each polypeptide chain set to an independent TLS group and all atomic *B*-factors set to the Wilson *B*-factor value of 81.5 Å^2^. This was followed by restrained positional and *B*-factor refinement (without prior phase information restraints).

Electron density for loops 265–291 (the RGD loop) and 349–354 of Prn-E were not visible, and these residues were not modeled. Except for these loops, no breaks in the main chain were evident for Mtd-P1 or Prn-E. No residues are in the disallowed region of the Ramachandran plot, with 81.7% (of the 2,170 residues other than glycine or proline) being in the most favored region and 17% in the additional allowed region. Molecular graphics were made using PyMol (http://pymol.sourceforge.net/).

### Coprecipitation assay.

For assays with Prn-E loop deletion mutants, 50 μL of His-tagged Prn-E, Prn-E(Δ190–199), and Prn-E(Δ399–407) (8 μM) were incubated (5 min, 25 °C) with 30 μl of Ni^2+^-nitrilotriacetic (NTA) agarose beads (Sigma). For preparation of beads, 50 μL of Ni^2+^-NTA agarose bead slurry (50% suspension in 30% ethanol) was centrifuged, and 20 μL of the overlying solution was removed. The Prn-E constructs were then added to the beads, and unbound Prn-E was removed by centrifugation and removal of overlying solution. Mtd (60 μM trimer) was added to the protein–bead mixture, incubated for 5 min at 25 °C, and washed with binding buffer (150 mM NaCl and 50 mM Tris, pH 8.0) three times. Bound protein was eluted with binding buffer supplemented with 500 mM imidazole, then visualized by SDS-PAGE. The final wash was devoid of protein. For quantification of binding in the coprecipitation assay, the intensity of Mtd bound to His-tagged Prn-E, Prn-E(Δ190–199), or Prn-E(Δ399–407) was measured using Kodak 1D imaging software (version 3.5.0), and the intensity of Mtd bound in the absence of His-tagged Prn-E was taken as background and subtracted from this value.

Comparison of Mtd-P6 and Mtd-M1 affinity for Prn-E was carried out as above, except that varying concentrations of Mtd were incubated with 80 μM Prn-E (5 min, 25 °C) prior to addition of Ni^2+^-NTA agarose beads. Beads (30 μl) were then added, and samples were handled as above. Intensities of protein bands were quantified from Coomassie stained SDS-PAGE gels using Kodak 1D imaging software. A standard concentration curve of known Mtd quantities was used to ensure linearity of measurements. The apparent *K*_d_ of Mtd-M1 for Prn-E was estimated by identifying total concentrations of Mtd-P6 and Mtd-M1 that yield equivalent amounts of bound Mtd. The *K*_d_ for Mtd-P6, as determined by SPR, was then used in the Langmuir isotherm to calculate the concentrations of bound and free Mtd-P6. The concentration of bound Mtd-P6 was applied to Mtd-M1 in the Langmuir isotherm to determine its apparent *K*_d_. Two equivalent concentrations of Mtd-P6 and Mtd-M1 (3 and 25 μM Mtd-P6 and Mtd-M1, respectively, and 8 and 63 μM Mtd-P6 and Mtd-M1) were used to estimate an apparent *K*_d_ of ∼0.5 mM for Mtd-M1.

### Surface plasmon resonance

The SPR measurements were performed using a Biacore 3000 at 25 °C. Approximately 100 response units (RUs) of biotinylated Prn-E were immobilized on the surface of a streptavidin sensor chip in running buffer (150 mM NaCl, 50 mM Tris, pH 8.0, and 0.005% Tween 20); a streptavidin sensor chip without any immobilized protein was used as a blank. Mtd-P1, Mtd-M1, and Mtd-P6 proteins and BPP-1, BMP-1, BIP-1, and BPP-6 phage were injected in running buffer at 30 μl/min for 2 min with the data collection rate set to high. A 2 min wash delay was used for complete dissociation of Mtd from Prn-E and accurate fitting of dissociation data. The data were analyzed using BIA evaluation 3.0 software for nonlinear curve fitting using a single-site model. Global fitting was used to analyze association and dissociation curves, except for BMP-1, which could not be fit. Six concentrations were used for each global fit, and experiments were carried out in duplicate. Concentrations of Mtd-P1 ranged from ∼20 to ∼125 × 10^−9^ M; of Mtd-P6 from ∼250 × 10^−9^ to ∼1.5 × 10^−6^ M; of BPP-1 phage from ∼1.2 to ∼3.7 × 10^−15^ M; of BPP-6 phage from ∼1 to ∼2 × 10^−16^ M; and of BMP-1 phage from ∼3.5 to ∼8.50 × 10^−12^ M. No interactions were detected for Mtd-M1 in the concentration range from 21 × 10^−9^ to 2.1 × 10^−6^ M and for BIP-1 phage in the concentration range from 8.3 × 10^−16^ to 8.3 × 10^−15^ M. Concentrations of BMP-1 equivalent to those used for BPP-1 and BPP-6 (e.g., 3.68 × 10^−15^ M) did not yield detectable binding.

For SPR assays using phage, BPP-1(Δ*brt*), BPP-6(Δ*brt*), and BMP-1(Δ*brt*) were harvested from B. bronchiseptica RB50. Cultures containing induced phage were treated with chloroform and centrifuged twice (8,000*g*, 10 min) to remove cell debris. Phage were then concentrated by centrifugation (13,000*g*, 3 h) in Corex tubes, and pellets (which contain phage) were resuspended in 50 mM Tris, pH 7.5, 0.1 M NaCl, 8 mM MgSO_4_, and 0.01% gelatin. Phage then were concentrated further by ultrafiltration (Amicon, YM-30) into running buffer. The number of plaque forming units per unit volume was determined from this final phage stock (on the appropriate B. bronchiseptica background), and this value was converted to molarity using Avogadro's number.

BIA simulation was used to estimate a *K*_d_ limit for Mtd-M1. The estimation is based on the observations that Mtd-P6 produces a binding event with an *R*_MAX_ value of 66.6 RU and the baseline noise level of detection is 1 RU. If it is assumed that either the on-rate or the off-rate of Mtd-M1 is the same as that for Mtd-P6, then the fact that 2 μM Mtd-M1 yields no detectable binding signal is consistent with an apparent *K*_d_ of ≥450 μM.

BIA simulation also was used to estimate an apparent *K*_d_ limit for BMP-1. The off-rate of BMP-1 was set to that of BPP-6 (1.43 × 10^−3^ s^−1^), and the *R*_MAX_ value of BMP-1 was set to that of BPP-6 (2.96 × 10^6^ RU). The fact that a concentration of BMP-1 of 8.50 × 10^−12^ M produces a binding event of 10 RU is consistent with an apparent *K*_d_ of ∼0.5 μM.

### Phage binding to *Bordetella*.

BPP-1(Δ*brt*), BPP-6(Δ*brt*), and BMP-1(Δ*brt*) phage were prepared with slight modifications to the above protocol. BPP-1(Δ*brt*) and BPP-6(Δ*brt*) were harvested from bacterial cultures grown in Luria broth containing 40 mM MgSO_4_, and BPP-6(Δ*brt*) was concentrated by ultracentrifugation at 4 °C (100,000*g*, 3 h). Phage concentrations were determined by plaquing on the appropriate host, B. bronchiseptica RB53 (Bvg^+^) for BPP-1 and BPP-6 and B. bronchiseptica RB54 (Bvg^–^) for BMP-1. BPP-1 and BPP-6 at ∼10^9^ plaque forming units ml^−1^ were incubated with an excess of B. bronchiseptica RB53 and RB54 (3 × 10^9^ bacteria ml^−1^) for 5 min, and unbound phage were isolated by filtering through a 0.8 μm cutoff membrane. Concentrations of BMP-1 up to 1 × 10^9^ ml^−1^ were incubated with RB53, RB54, and E. coli TOP10, as above. Free (unbound) phage were quantified by plaquing on RB53 or RB54 B. bronchiseptica, as appropriate. The binding isotherms were fit by nonlinear regression to yield a *K*_d_.

### Quantification of bacterially associated pertactin.

The quantity of bacterially-associated Prn-E was determined by immunoblotting, using known concentrations of purified Prn-E as a standard. Overnight cultures of B. bronchiseptica RB53 (Bvg^+^) and RB54 (Bvg^–^) were pelleted by centrifugation and washed twice with wash buffer (150 mM NaCl and 50 mM Tris, pH 8.0), then resuspended in wash buffer to a final *A*_600_ of 1.0 (equivalent to 3.3 × 10^9^ bacteria ml^−1^). Approximately 10 μl of each culture were lysed by boiling in SDS-PAGE sample buffer, separated on by 10% SDS-PAGE, and visualized by western blot using PeM19 as the primary antibody and an anti-mouse horseradish peroxidase conjugate as the secondary antibody. PeM19 is a mouse monoclonal antibody that recognizes the linear epitope RELSA on Prn-E [40]. Fluorescence from blots was quantified with a Typhoon 8600 (Amersham Pharmacia) imaging scanner and confirmed to be within the linear range of detection. These measurements yield a value of 3,000 molecules of pertactin per bacterium.

The approximate surface area of a Gram-negative bacterium is 6 × 10^−6^ mm^2^, resulting in a surface density for pertactin of 8.26 × 10^−16^ mol mm^−2^. The surface density of Prn-E on the SPR chip is 1.67 × 10^−15^ mol mm^−2^, as determined by the 100 RU of Prn-E bound to the chip.

## Supporting Information

Figure S1 Receptor-Binding Sites of Mtd The three independent receptor-binding sites of trimeric Mtd are denoted by dotted lines. The view is looking at the base of the pyramid-shaped Mtd trimer. Individual Mtd protomers of unbound Mtd-P1 are depicted in ribbon representation in blue, red, and gold. The β2β3 loop of one protomer contributes invariant residues Tyr322 and Tyr333 to the binding site of a neighboring protomer. Side chains of variable residues are shown, and the trimer axis is indicated by the black triangle in the middle.
(1.9 MB PDF)

Figure S2 Conformations of Major and Minor Pertactin Loops (A and B) The conformations of the (A) major and (B) minor loops from B. bronchiseptica pertactin bound to Mtd are in yellow stick representation, and the conformations of these same loops from free B. pertussis pertactin [12] are in purple stick representation. Sequence alignment between B. bronchiseptica (*B.b.*) and B. pertussis (*B.p.*) pertactin in this region is shown below. The superposition is based on 492 Cα atoms of bound B. bronchiseptica pertactin and unbound B. pertussis pertactin (root-mean-square deviation 0.83 Å).
(1.2 MB PDF)

Figure S3 Molecular Surface Representations of Receptor-Binding Sites of Mtd Variants (A) The molecular surface of Mtd-M1, as experimentally determined [7], is shown (green, hydrophobic residues; red, hydrophilic residues), with underlying backbone in gray and side chain carbons, oxygens, and nitrogens in gray, red, and blue, respectively. Mtd residues are labeled in blue.
(B) The molecular surface of Mtd-P6, as modeled based on Mtd-M1. The five Mtd-P6 residues differing from Mtd-M1 were modeled in rotamer conformations that avoided steric clashes with neighboring atoms.
(978 KB PDF)

Figure S4 Fitting Residuals for Surface Plasmon Resonance Sensorgrams (A) Residuals for fitting of Mtd-P1. Residuals for fits to the association phase occur between 0 and 120 s, and residuals for fits to the dissociation phase occur between 120 and 250 s. Gaps between residuals represent data that was not included in fitting.
(B) Residuals for fitting of Mtd-P6.
(C) Residuals for fitting of BPP-1.
(D) Residuals for fitting of BPP-6.
(1.0 MB PDF)

Figure S5 Association of Mtd-P6 and Mtd-M1 with Pertactin Coprecipitation of varying concentrations of Mtd-P6 and Mtd-M1 (total trimer concentrations indicated above lanes) with 80 μM Prn-E using Ni^2+^-NTA beads and visualized by SDS-PAGE and Coomassie staining. Concentrations of Mtd·Prn-E complexes are equivalent when 3 μM Mtd-P6 is incubated with Prn-E as when 25 μM Mtd-M1 is incubated with Prn-E, and when 8 μM Mtd-P6 is incubated with Prn-E as when 63 μM Mtd-M1 is incubated with Prn-E.
(531 KB PDF)

Figure S6 Quantification of *Bordetella*-Associated Pertactin Western blot probed with the anti-pertactin monoclonal antibody PeM19 of known quantities of purified Prn-E (50, 25, 12, 6, and 1 ng), 10 μL of an *A*_600_ = 1 culture of B. bronchiseptica RB54, and 10, 5, and 2.5 μL of an *A*_600_ = 1 culture of B. bronchiseptica RB54. Graph below shows linearity of secondary antibody fluorescence in detection of Prn-E standards (with 50 ng omitted due to nonlinearity).
(265 KB PDF)

Figure S7 Phage Binding to *Bordetella*
Binding of BMP-1 to Bvg^–^ *Bordetella*. While the identity and number of receptors is unknown, the data can be fit with a single-site binding model that yields a *K*_d_ of 2.7 × 10^−13^ M. Binding to E. coli was subtracted as nonspecific background.
(116 KB PDF)

Table S1 Data Collection and Refinement Statistics (Molecular Replacement) (76 KB PDF)

Table S2 Mtd-P1 Residues Buried by Contact with Prn-E (45 KB PDF)

Table S3 Prn-E Residues Buried by Contact with Mtd-P1 (48 KB PDF)

### Accession Numbers

Accession numbers for genes mentioned in this paper from the National Center for Biotechnology Information (http://www.ncbi.nlm.nih.gov) are: *Mtd* (NP_958677.1) and *Prn-E* (NP_887912).

The structure coordinates of Mtd-P1 (PDB ID 1YU0) from the Protein Data Bank (PDB) (http://www.rcsb.org/pdb) were used for model building via molecular replacement. The structure coordinates of Mtd-P1·Prn-E determined in this study are deposited as PDB ID 2IOU.

## Abbreviations

DGR: diversity-generating retroelement
Mtd: major tropism determinant
NTA: nitrilotriacetic
Prn-E: ectodomain of pertactin
RU: response unit
SC: surface complementarity
SPR: surface plasmon resonance
TCR: T cell receptor

## References

[pbio-0060131-b001] Pancer Z, Cooper MD (2006). The evolution of adaptive immunity. Annu Rev Immunol.

[pbio-0060131-b002] Davis MM, Bjorkman PJ (1988). T-cell antigen receptor genes and T-cell recognition. Nature.

[pbio-0060131-b003] Alder MN, Rogozin IB, Iyer LM, Glazko GV, Cooper MD (2005). Diversity and function of adaptive immune receptors in a jawless vertebrate. Science.

[pbio-0060131-b004] Liu M, Deora R, Doulatov SR, Gingery M, Eiserling FA (2002). Reverse transcriptase-mediated tropism switching in Bordetella bacteriophage. Science.

[pbio-0060131-b005] Doulatov S, Hodes A, Dai L, Mandhana N, Liu M (2004). Tropism switching in Bordetella bacteriophage defines a family of diversity-generating retroelements. Nature.

[pbio-0060131-b006] Liu M, Gingery M, Doulatov SR, Liu Y, Hodes A (2004). Genomic and genetic analysis of Bordetella bacteriophages encoding reverse transcriptase-mediated tropism-switching cassettes. J Bacteriol.

[pbio-0060131-b007] McMahon SA, Miller JL, Lawton JA, Kerkow DE, Hodes A (2005). The C-type lectin fold as an evolutionary solution for massive sequence variation. Nat Struct Mol Biol.

[pbio-0060131-b008] Uhl MA, Miller JF (1996). Integration of multiple domains in a two-component sensor protein: the Bordetella pertussis BvgAS phosphorelay. EMBO J.

[pbio-0060131-b009] Cummings CA, Bootsma HJ, Relman DA, Miller JF (2006). Species- and strain-specific control of a complex, flexible regulon by Bordetella BvgAS. J Bacteriol.

[pbio-0060131-b010] Akerley BJ, Cotter PA, Miller JF (1995). Ectopic expression of the flagellar regulon alters development of the Bordetella-host interaction. Cell.

[pbio-0060131-b011] Cotter PA, Miller JF (1997). A mutation in the Bordetella bronchiseptica bvgS gene results in reduced virulence and increased resistance to starvation, and identifies a new class of Bvg-regulated antigens. Mol Microbiol.

[pbio-0060131-b012] Emsley P, Charles IG, Fairweather NF, Isaacs NW (1996). Structure of Bordetella pertussis virulence factor P.69 pertactin. Nature.

[pbio-0060131-b013] Mattoo S, Cherry JD (2005). Molecular pathogenesis, epidemiology, and clinical manifestations of respiratory infections due to Bordetella pertussis and other Bordetella subspecies. Clin Microbiol Rev.

[pbio-0060131-b014] Li J, Fairweather NF, Novotny P, Dougan G, Charles IG (1992). Cloning, nucleotide sequence and heterologous expression of the protective outer-membrane protein P.68 pertactin from Bordetella bronchiseptica. J Gen Microbiol.

[pbio-0060131-b015] Charles I, Fairweather N, Pickard D, Beesley J, Anderson R (1994). Expression of the Bordetella pertussis P.69 pertactin adhesin in Escherichia coli: fate of the carboxy-terminal domain. Microbiology.

[pbio-0060131-b016] Lo Conte L, Chothia C, Janin J (1999). The atomic structure of protein–protein recognition sites. J Mol Biol.

[pbio-0060131-b017] MacCallum RM, Martin AC, Thornton JM (1996). Antibody–antigen interactions: contact analysis and binding site topography. J Mol Biol.

[pbio-0060131-b018] Fellouse FA, Wiesmann C, Sidhu SS (2004). Synthetic antibodies from a four-amino-acid code: a dominant role for tyrosine in antigen recognition. Proc Natl Acad Sci U S A.

[pbio-0060131-b019] Mooi FR, van Oirschot H, Heuvelman K, van der Heide HG, Gaastra W (1998). Polymorphism in the Bordetella pertussis virulence factors P.69/pertactin and pertussis toxin in The Netherlands: temporal trends and evidence for vaccine-driven evolution. Infect Immun.

[pbio-0060131-b020] Lawrence MC, Colman PM (1993). Shape complementarity at protein/protein interfaces. J Mol Biol.

[pbio-0060131-b021] Mammen M, Choi S, Whitesides GM (1998). Polyvalent interactions in biological systems: implications for design and use of multivalent ligands and inhibitors. Angew Chem Int Ed Engl.

[pbio-0060131-b022] Barton ES, Connolly JL, Forrest JC, Chappell JD, Dermody TS (2001). Utilization of sialic acid as a coreceptor enhances reovirus attachment by multistep adhesion strengthening. J Biol Chem.

[pbio-0060131-b023] James LC, Roversi P, Tawfik DS (2003). Antibody multispecificity mediated by conformational diversity. Science.

[pbio-0060131-b024] Mueller CM, Jemmerson R (1996). Maturation of the antibody response to the major epitope on the self antigen mouse cytochrome c. Restricted V gene usage, selected mutations, and increased affinity. J Immunol.

[pbio-0060131-b025] Batista FD, Neuberger MS (1998). Affinity dependence of the B cell response to antigen: a threshold, a ceiling, and the importance of off-rate. Immunity.

[pbio-0060131-b026] Foote J, Milstein C (1991). Kinetic maturation of an immune response. Nature.

[pbio-0060131-b027] Bankovich AJ, Raunser S, Juo ZS, Walz T, Davis MM (2007). Structural insight into pre-B cell receptor function. Science.

[pbio-0060131-b028] van der Merwe PA, Davis SJ (2003). Molecular interactions mediating T cell antigen recognition. Annu Rev Immunol.

[pbio-0060131-b029] Bachmann MF, Kalinke U, Althage A, Freer G, Burkhart C (1997). The role of antibody concentration and avidity in antiviral protection. Science.

[pbio-0060131-b030] Hornick CL, Karush F (1972). Antibody affinity. 3. The role of multivalance. Immunochemistry.

[pbio-0060131-b031] Garcia KC, Scott CA, Brunmark A, Carbone FR, Peterson PA (1996). CD8 enhances formation of stable T-cell receptor/MHC class I molecule complexes. Nature.

[pbio-0060131-b032] Schamel WW, Arechaga I, Risueno RM, van Santen HM, Cabezas P (2005). Coexistence of multivalent and monovalent TCRs explains high sensitivity and wide range of response. J Exp Med.

[pbio-0060131-b033] Boniface JJ, Rabinowitz JD, Wulfing C, Hampl J, Reich Z (1998). Initiation of signal transduction through the T cell receptor requires the multivalent engagement of peptide/MHC ligands. Immunity.

[pbio-0060131-b034] McKeithan TW (1995). Kinetic proofreading in T-cell receptor signal transduction. Proc Natl Acad Sci U S A.

[pbio-0060131-b035] Cotter PA, Miller JF (1994). BvgAS-mediated signal transduction: analysis of phase-locked regulatory mutants of Bordetella bronchiseptica in a rabbit model. Infect Immun.

[pbio-0060131-b036] Higuchi R, Krummel B, Saiki RK (1988). A general method of in vitro preparation and specific mutagenesis of DNA fragments: study of protein and DNA interactions. Nucleic Acids Res.

[pbio-0060131-b037] Otwinowski ZM W (1997). Processing of X-ray diffraction data collected in oscillation mode. Methods Enzymol.

[pbio-0060131-b038] Winn MD (2003). An overview of the CCP4 project in protein crystallography: an example of a collaborative project. J Synchrotron Radiat.

[pbio-0060131-b039] Jones TA, Zou JY, Cowan SW, Kjeldgaard (1991). Improved methods for building protein models in electron density maps and the location of errors in these models. Acta Crystallogr A.

[pbio-0060131-b040] Hijnen M, Mooi FR, van Gageldonk PG, Hoogerhout P, King AJ (2004). Epitope structure of the Bordetella pertussis protein P.69 pertactin, a major vaccine component and protective antigen. Infect Immun.

