# Supplementary material for: Selective Ligand Recognition by a Diversity-Generating Retroelement Variable Protein
Source: PLoS Biol. 2008 Jun 3;6(6):e131. doi: 10.1371/journal.pbio.0060131 (PMC2408619; doi:10.1371/journal.pbio.0060131)
Supplement: Supplementary file 2 [file pbio.0060131.sg002.pdf]

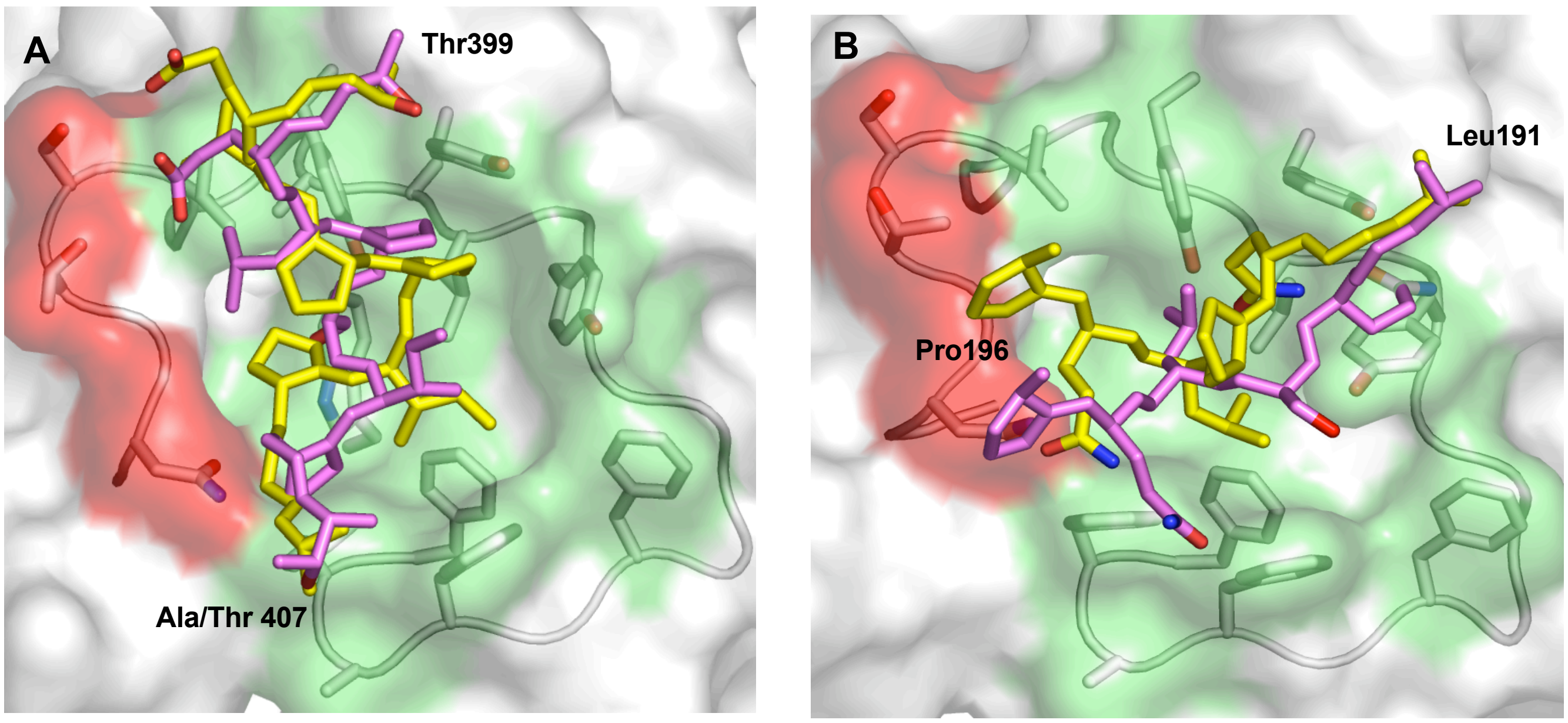


```
B.b  399 TELPPIPGA
         :::: :::
B.p. 399 TELPSIPGT
```

```
B.b  190 TLQPLQPEDL
           :: ::::::
B.p. 190 ALQSLQPEDL
```
