# Supplementary material for: Selective Ligand Recognition by a Diversity-Generating Retroelement Variable Protein
Source: PLoS Biol. 2008 Jun 3;6(6):e131. doi: 10.1371/journal.pbio.0060131 (PMC2408619; doi:10.1371/journal.pbio.0060131)
Supplement: Supplementary file 3 [file pbio.0060131.sg003.pdf]

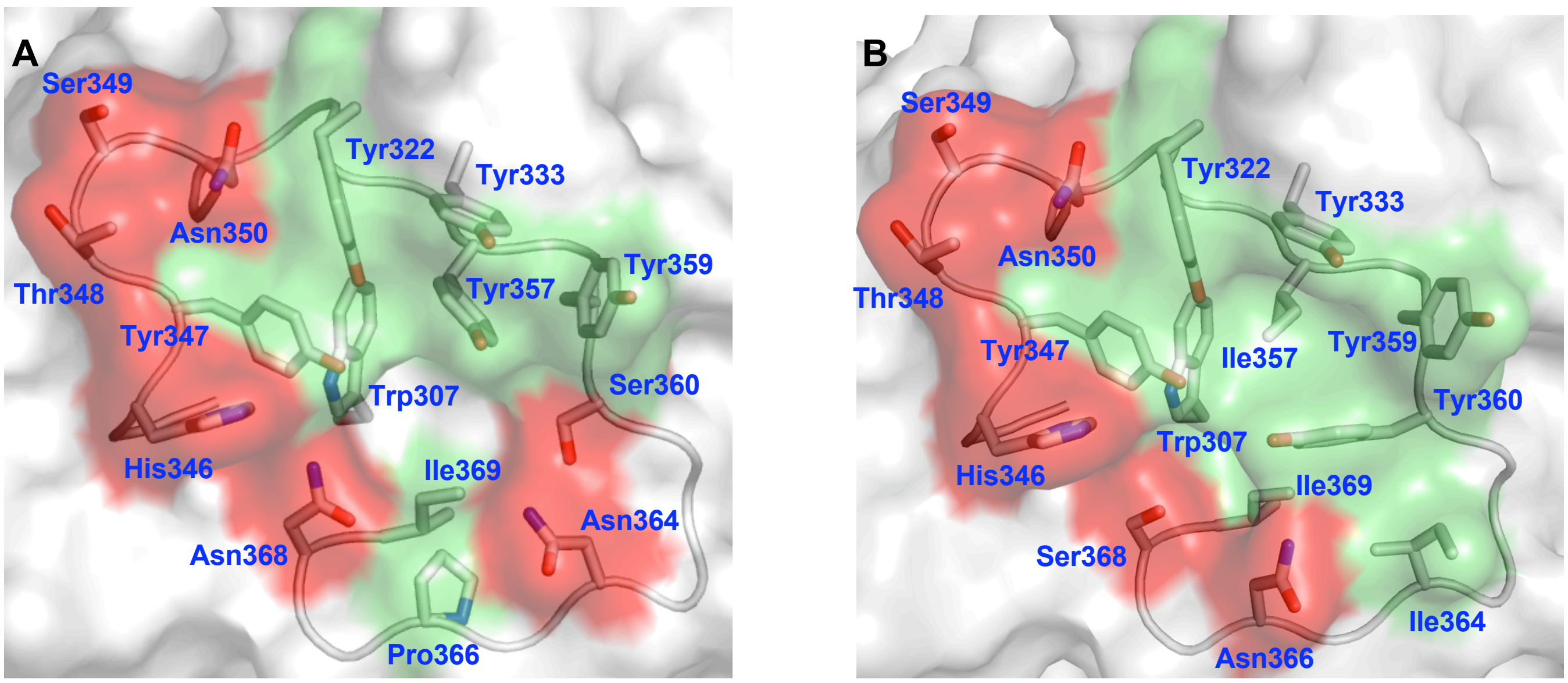

Mtd-M1

Mtd-P6 (modeled)
