# Supplementary material for: Selective Ligand Recognition by a Diversity-Generating Retroelement Variable Protein
Source: PLoS Biol. 2008 Jun 3;6(6):e131. doi: 10.1371/journal.pbio.0060131 (PMC2408619; doi:10.1371/journal.pbio.0060131)
Supplement: Supplementary file 5 [file pbio.0060131.sg005.pdf]

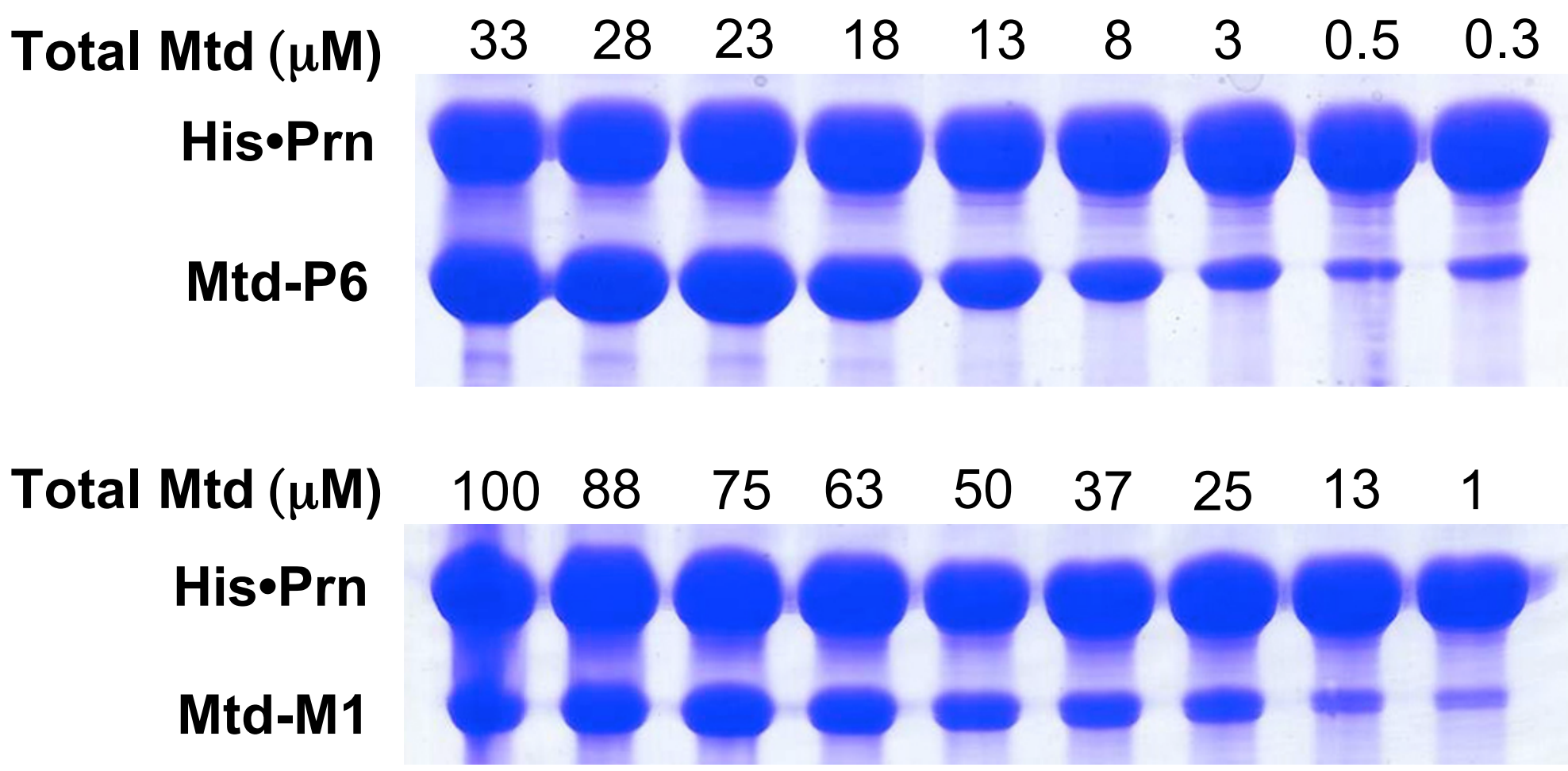
Total Mtd (μM)
33 28 23 18 13 8 3 0.5 0.3
His•Prn
Mtd-P6
Total Mtd (μM)
100 88 75 63 50 37 25 13 1
His•Prn
Mtd-M1
