# Supplementary material for: Selective Ligand Recognition by a Diversity-Generating Retroelement Variable Protein
Source: PLoS Biol. 2008 Jun 3;6(6):e131. doi: 10.1371/journal.pbio.0060131 (PMC2408619; doi:10.1371/journal.pbio.0060131)
Supplement: Supplementary file 7 [file pbio.0060131.sg007.pdf]

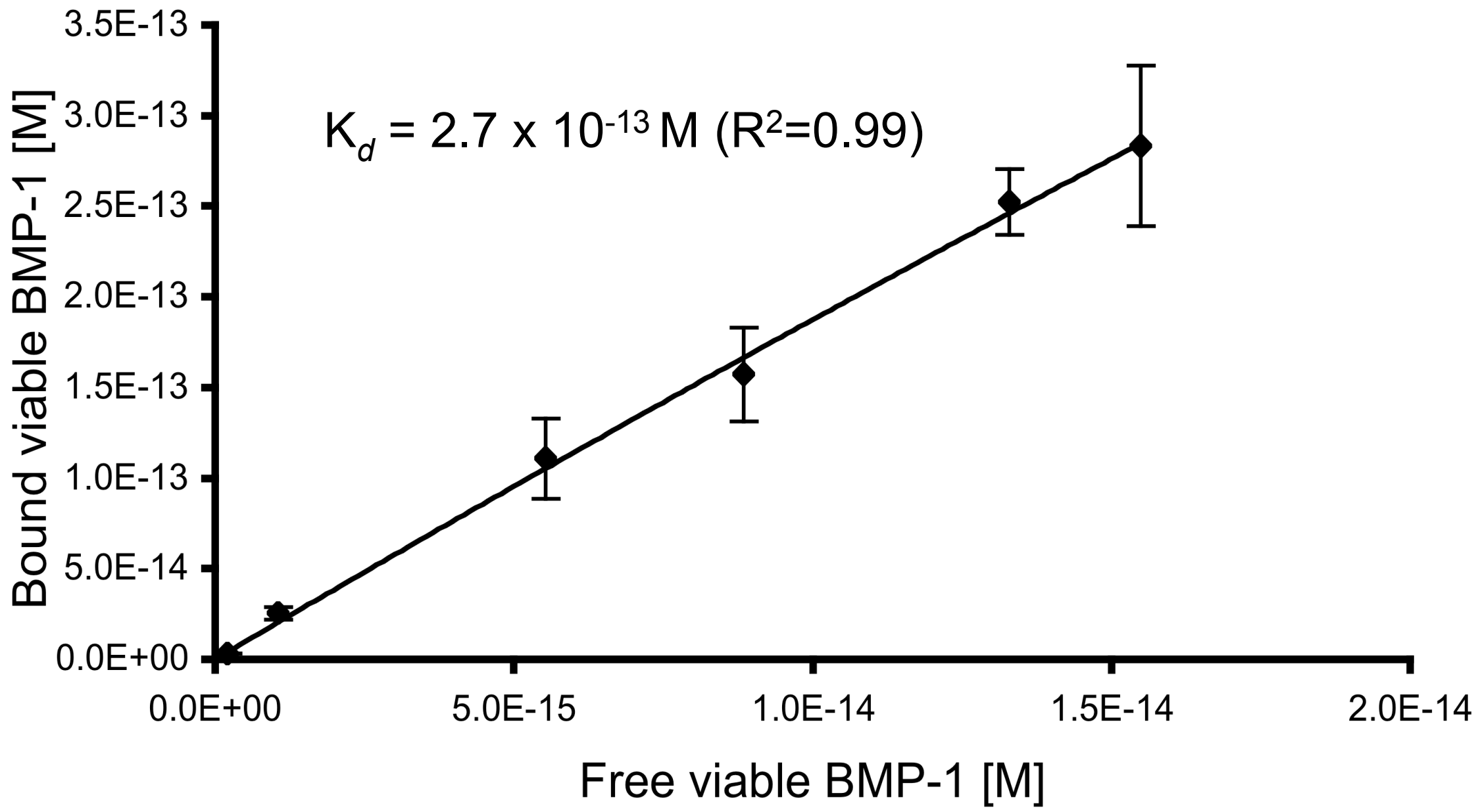

K_d = 2.7 x 10^{-13} M (R^2=0.99)
Bound viable BMP-1 [M]
3.5E-13
3.0E-13
2.5E-13
2.0E-13
1.5E-13
1.0E-13
5.0E-14
0.0E+00
0.0E+00
5.0E-15
1.0E-14
1.5E-14
2.0E-14
Free viable BMP-1 [M]
