# Supplementary material for: Selective Ligand Recognition by a Diversity-Generating Retroelement Variable Protein
Source: PLoS Biol. 2008 Jun 3;6(6):e131. doi: 10.1371/journal.pbio.0060131 (PMC2408619; doi:10.1371/journal.pbio.0060131)
Supplement: Supplementary file 8 [file pbio.0060131.st001.pdf]

Table S1. Data collection and refinement statistics (Molecular Replacement)

	Mtd-P1•Prn-E[a]
**Data collection**	
Space group	$P6_1$
Cell dimensions	
a, *c* (Å)	413.5, 98.9
Resolution (Å)	50.00-3.16 (3.26-3.16)[b]
R_{merge}[c]	15.6 (78.7)
$I / \sigma I$	13.8 (2.0)
Completeness (%)	100.0 (100.0)
Redundancy	9.2 (8.3)
**Refinement**	
Resolution (Å)	49.63-3.16 (3.24-3.16)
No. reflections	157,861
R_{work} / R_{free}[d]	22.4 / 24.9 (33.0 / 36.0)
No. atoms	
Protein	23,896
Ligand/ion	4
Water	0
B-factors (Å^2)	
Protein	69.5
Ligand/ion	52.3
Water	0
R.m.s. deviations	
Bond lengths (Å)	0.008
Bond angles (°)	1.141
Estimated coordinate error (Å)[e]	0.312, 0.246

[a]Data collected from single crystal.

[b]Highest resolution shell is shown in parenthesis.

[c]$R_{merge} = 100 \times \frac{\sum_h \sum_i |I_{h,i} - I_h|}{\sum_h \sum_i I_{h,i}}$, where I_h is the mean intensity of symmetry-related reflections, $I_{h,i}$.

[d]$R = 100 \times \frac{\sum_{hkl} |F_{obs} - F_{calc}|}{\sum_{hkl} F_{obs}}$, where R_{free} is calculated for a randomly chosen 5 % of reflections (F > 0) omitted from refinement, and R_{work} is calculated for the remaining 95 % of reflections (F > 0) included in refinement.

[e]First value based on R_{free}, and second on maximum likelihood.
