# Supplementary material for: Selective Ligand Recognition by a Diversity-Generating Retroelement Variable Protein
Source: PLoS Biol. 2008 Jun 3;6(6):e131. doi: 10.1371/journal.pbio.0060131 (PMC2408619; doi:10.1371/journal.pbio.0060131)
Supplement: Supplementary file 9 [file pbio.0060131.st002.pdf]

Table S2. Mtd-P1 residues buried by contact with Prn-E

Mtd residue (Chains)	Buried surface area ($Å^2$)[a]	Pertactin loop contacted[b]	Contact[c]
Tyr359 (B, D)[d]	92.0	Major	+
Tyr322 (C, E)	56.5	Major	+
Phe368 (B, D)	48.2	Major	+
Phe366 (B, D)	45.4	Major	+
Ala319 (B, D)	43.7	Minor	+
Tyr333 (C, E)	42.6	Major	+
Glu321 (C, E)	41.8	Major	+
Tyr333 (B, D)	40.3	Minor	+
Asn346 (B, D)	37.3	Major	+
Asn346 (A, F)	37.2	Minor	+
Phe366 (A, F)	36.2	Minor	+
Phe364 (B, D)	35.0		—
Ser320 (C, E)	34.0	Major	+
Leu350 (B, D)	33.5		—
Tyr359 (A, F)	32.1	Minor	+
Glu321 (B, D)	31.1	Minor	+
Glu321 (A, F)	30.8		—
Tyr322 (B, D)	29.8	Minor	+
Val316 (B, D)	28.4	Minor	+
Phe368 (A, F)	28.1	Minor	+
Ser320 (B, D)	24.2		—
Leu357 (B, D)	19.4	Major	+
Leu350 (A, F)	18.5	Minor	+
Asn317 (B, D)	18.2	Minor	+
Ser320 (A, F)	16.6		—
Thr348 (A, F)	15.8	Minor	+
Gly318 (B, D)	15.4	Minor	+
Leu357 (A, F)	14.6	Minor	+
Thr323 (B, D)	13.3		—
Phe369 (B, D)	9.8		—
Phe369 (A, F)	8.3	Minor	+
Gly347 (B, D)	5.3		—
Trp307 (A, F)	4.0		—
Ala324 (A, F)	2.6		—
Ser360 (B, D)	2.5	Major	+
Gly347 (A, F)	0.8		—

[a]The average buried surface area of the two Mtd-P1/Prn-E complexes in the asymmetric unit of the crystal is reported. The probe radius used was 1.4 Å.
[b]The major pertactin loop is composed of residues 399-407 and the minor pertactin loop of residues 190-199. The pertactin loop is indicated if it is contacted (≤ 4 Å) by the Mtd residue.
[c] + denotes that residue is ≤ 4 Å of pertactin. — denotes that residue is > 4 Å of pertactin.
[d]Residues in blue are variable.
