# Supplementary material for: Selective Ligand Recognition by a Diversity-Generating Retroelement Variable Protein
Source: PLoS Biol. 2008 Jun 3;6(6):e131. doi: 10.1371/journal.pbio.0060131 (PMC2408619; doi:10.1371/journal.pbio.0060131)
Supplement: Supplementary file 10 [file pbio.0060131.st003.pdf]

Table S3. Prn-E residues buried by contact with Mtd-P1

Pertactin residue	Buried surface area (Å^2)[a]	Mtd Binding Site[b]	Contact[c]
Leu194	137.0	Minor	+
Ile404	117.0	Major	+
Pro405	101.0	Major	+
Pro403	85.1	Major	+
Thr399	84.3	Major	+
Gln192	82.2	Minor	+
Ala348	70.5	Minor	+
Leu401	70.1		—
Arg242	55.1		—
Arg350	40.3	Major	+
Leu191	37.9		—
Pro196	36.8		—
Asp250	35.2	Minor	+
Arg323	33.2	Minor	+
Ala407	32.9		—
Gln195	29.6		—
Val377	26.2		—
Gly347	24.1	Minor	+
Pro379	21.4	Major	+
Gly406	18.4	Major	+
Phe351	13.3		—
Arg349	12.9		—
Arg110	10.6		—
Val109	9.8		—
Pro402	7.5	Major	+
Tyr375	5.9		—
Gly346	5.5		—
Met249	3.4		—
Gly218	3.0		—

[a]The average buried surface area of the two Mtd-P1/Prn-E complexes in the asymmetric unit of the crystal is reported.
[b]The Mtd binding site is indicated if it is contacted (≤ 4 Å) by the pertactin residue.
The major Mtd the site is composed of variable residues from chain B and conserved residues from the β2β3 (residues 316-333) loop of chain C in one of the Mtd-P1/Prn-E complexes in the asymmetric unit of the crystal. In the other complex in the asymmetric unit, the major Mtd site is composed of variable residues from chain D and conserved residues from the β2β3 loop of chain E. The minor Mtd site is composed of variable residues from chain A and conserved residues from the β2β3 loop of chain B in one complex, and variable residues from chain F and conserved residues from the β2β3 loop of chain D in the other complex. The Mtd binding sites formed by variable residues of chains C and E are unoccupied.
[c] + denotes that residue is ≤ 4 Å of pertactin. — denotes that residue is > 4 Å of pertactin.
